# Complete genome sequence of *Janthinobacterium lividum* EIF5 isolated from soil of a temperate broadleaf and mixed forest

**DOI:** 10.1128/mra.00377-26

**Published:** 2026-05-29

**Authors:** Ines Friedrich, Jana Wagner, Anja Poehlein, Rolf Daniel

**Affiliations:** 1 Department of Genomic and Applied Microbiology, University of Göttingen 9375, Göttingen, Germany; University of Manitoba, Winnipeg, Canada

**Keywords:** *Janthinobacterium lividum*, forest soil, violacein

## Abstract

We report the complete genome sequence of *Janthinobacterium lividum* EIF5, isolated from soil of a temperate broadleaf and mixed forest in Osterode am Harz, Germany. The genome consists of a 6,307,211 bp chromosome and a 225,743-bp plasmid with GC contents of 62.69% and 54.77%, respectively.

## ANNOUNCEMENT

Members of the genus *Janthinobacterium* are commonly found in soil and are characterized by the production of the antimicrobial pigment violacein (1). To expand genomic resources for this genus, we sequenced the genome of *J. lividum* EIF5, isolated from soil of a temperate mixed forest collected near Osterode am Harz, Germany (51°44′17.5″ N, 10°15′51.5″ E), on 12 December 2023. Isolation of the strain was performed using a modified Ryall and Moss medium (2). The medium consisted of beef extract (0.25 g/L), yeast extract (0.5 g/L), peptone (1.25 g/L), NaCl (1.25 g/L), and agar (20 g/L). After autoclaving and cooling to approximately 45°C, filter-sterilized polymyxin B (15 µg/mL), sodium deoxycholate (300 µg/mL), and nystatin (50 µg/mL) were added. Soil samples were suspended by diluting 1 g of soil in 20 mL of 0.9% NaCl solution. Subsequently, 100 µL aliquots were spread on plates. After 2 days of incubation at 25°C, a violet-pigmented colony, indicative of violacein production, was transferred to R2A agar (3) and restreaked at least four times at 25°C to obtain a pure culture. The isolate was selected for genome sequencing due to the ecological and antimicrobial relevance of violacein-producing bacteria. Preliminary taxonomic identification based on 16S rRNA gene sequence analysis revealed 99.93% identity to *Janthinobacterium lividum* strain SQ66 (GenBank accession no. KC920976.1). High-molecular-weight genomic DNA was extracted using the NucleoMag DNA Bacteria Kit (Macherey-Nagel, Düren, Germany), according to the manufacturer’s instructions. For Nanopore sequencing, 600 ng of genomic DNA was used for library preparation with the Native Barcoding Kit 24 V14 (SQK-NBD114.24; barcode 15) as recommended by the manufacturer (Oxford Nanopore Technologies, Oxford, UK). Genomic DNA was neither sheared nor size selected prior to library preparation. Sequencing was performed for 113 h by using a MinION Mk1B device, MinKNOW software v25.05.12, and an R10.4.1 flow cell. All software was used with default settings unless otherwise stated. Base calling and demultiplexing were performed using Dorado v1.2.0 (SUP model). Sequencing generated 188,485 reads comprising 1,396,446,166 bp, with a read N50 of 11,152 bp. Raw reads were filtered using Filtlong v0.3.1 (4) to retain high-quality reads longer than 1 kb. Filtered reads were subsampled using Trycycler v0.5.5 (5) based on an estimated genome size of 6 Mb. Independent assemblies were generated with Flye v2.9.6 (6), Raven v1.8.3 (7), and Miniasm v0.3 (8) with Minipolish v0.1.3 (8). Assemblies were combined using Trycycler, and the consensus assembly was polished with Medaka v2.1.0 (Oxford Nanopore Technologies). Circularization was confirmed during assembly and consensus generation, where overlapping contig ends were identified and trimmed to produce complete circular sequences for both chromosome and plasmid. Reads were mapped back to the final assembly using Minimap2 v2.29 (9). Mean coverage was calculated using SAMtools v1.22 (10), yielding an average genome coverage of 211-fold. The final assembly consists of a circular chromosome (6,307,211 bp; GC content 62.69%) and a plasmid (225,743 bp; GC content 54.77%). The genome was reoriented with Dnaapler v1.2.0 (11). Genome annotation was performed using the Prokaryotic Genome Annotation Pipeline v6.10 (12), and genome quality was assessed using CheckM2 v1.1.0 (13), as summarized in Table 1.

**TABLE 1:** General features of the genome of *Janthinobacterium lividum* EIF5 (accession: JBWBFR000000000) based on PGAP annotation (12) and CheckM2 analysis (13)

Feature	Chromosome	Plasmid
Size (bp)	6,307,211	225,743
GC content (%)	62.69	54.77
Total number of genes	5,653	150
Protein-coding sequences (CDS)	5,532	150
rRNA genes (5S/16S/23S)	9/8/8	0/0/0
tRNA genes	92	0
ncRNA genes	3	0
Pseudogenes	36	0
Genome completeness	100%	–
Genome contamination	0.07%	–

**Fig 1:**
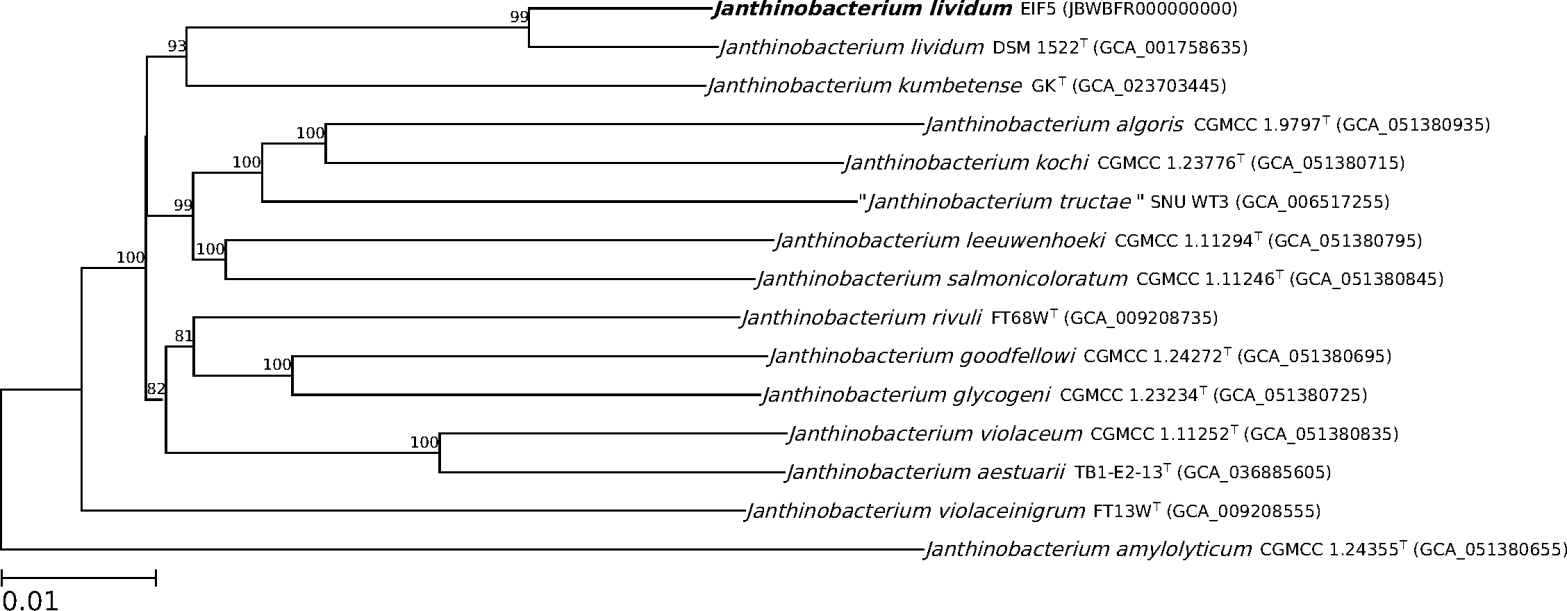
Whole-genome-based phylogenetic tree showing the position of *Janthinobacterium lividum* EIF5 (bold) among closely related *Janthinobacterium* type strains. The tree was inferred by using TYGS (14). The tree was calculated from GBDP distances using FastME with 100 pseudo-bootstrap replicates (15). The tree was visualized using ETE 3 (16) and rooted at the midpoint.

Whole-genome-based phylogeny of strain EIF5 was inferred using the Type (Strain) Genome Server (TYGS (14; accessed 12 March 2026). The digital DNA-DNA hybridization (dDDH) value between EIF5 and *Janthinobacterium lividum* DSM 1522^T^ was 79.0%, calculated using formula d4, exceeding the species delineation threshold of 70%. Thus, EIF5 represents a new strain within the species *J. lividum* (Fig. 1).

## Data Availability

The complete genome sequence has been deposited in DDBJ/ENA/GenBank under accession number JBWBFR000000000. Raw Nanopore reads are available in the NCBI Sequence Read Archive under accession number SRR37584688.
